# Treatment of Tuberculous Bone Disease

**Published:** 1915-08-28

**Authors:** 


### THE ALTON HOSPITAL FOR CRIPPLES.

### Treatment of Tuberculous Bone Disease.

The hospital for crippled children at Alton is not one of those institutions of the existence of which the public has been left in ignorance. It will come, therefore, as something of a surprise to find that the interesting report which has lately been issued is the first medical report that has been circulated since the opening of the hospital in 1908. This illustrated pamphlet gives a very fair idea of the scope of the work done at Alton; and from the photographs alone the public will be able in some degree to judge of the thoroughness with which the treatment of tuberculous bone disease is being undertaken. One picture shows a band of children setting out for a Nature-study lesson while receiving "sun bath" treatment. The practice of heliotherapy is one of the important features of the Alton treatment, and is carried out to the fullest extent. Deficiency of sunshine is supplemented on occasion by the use of the ultra-violet rays.

Needless to say, this hospital uses large quantities of plaster for spinal jackets and other splints, but the employment of celluloid has also received attention at this institution. By the addition of calcium chloride to the celluloid solution the disadvantage of inflammability has been overcome. Since September 1908 up to the end of last March 1,267 patients were admitted, and it is interesting to note that the cases are treated practically to a finish, the average duration of stay being about 400 days. The spinal cases stayed on an average 491 days.

This length of stay makes some provision for the education of the children a matter of urgent concern, and a school has been organised which is now certified by, and under the direct supervision of, the Board of Education. Hand-work plays a prominent part in this school, and in this way the brain is constantly and naturally developed through this "tutor." The report, which has been written by Dr. Gauvain, M.A., M.R.C.S., contains many instructive details.

One other point may be referred to as worthy of record: on no occasion has it been necessary to perform any abdominal operation. Freedom from such a complication as appendicitis is somewhat remarkable, but it is a bold presumption to attribute this freedom to the "plain and regular diet of the patients and the careful attention given to the bowels." It would be interesting to know if any of our authorities on tuberculosis have claimed to note an antagonism between the causes of appendicitis and the bacillus of tuberculosis.

Tuberculosis disease causes perhaps more crippling than any other. The mortality itself is high, and is contributed to by the lack of facilities for prolonged and systematic treatment, such as general hospitals cannot be called upon to undertake. It was to remedy this defect that this hospital was founded. Conservative treatment is naturally the aim which the Alton authorities have in view, and this requires not only prolonged residence and complicated splinting or mechanical assistance, but also efficient after-care and guidance when the patient is discharged.

### After-care.

The fullest instructions for the after-care of each patient are sent to the parents on discharge, and, when possible, the patients are seen at regular intervals at the out-patient department in London. In this way many cripples have been converted from dependants to wage-earners and producers.

